# Association of resection margin distance with anastomotic recurrence in stage I-III colon cancer: data from the National Colorectal Cancer Cohort (NCRCC) study in China

**DOI:** 10.1007/s00384-024-04684-x

**Published:** 2024-07-12

**Authors:** Fei Huang, Shan Jiang, Ran Wei, Tixian Xiao, Fangze Wei, Zhaoxu Zheng, Qian Liu

**Affiliations:** 1 Department of Colorectal Surgery, National Cancer Center/National Clinical Research Center for Cancer/Cancer Hospital, Chinese Academy of Medical Sciences and Peking Union Medical College, 17 Panjiayuan Nanli, Chaoyang District, Beijing, 100021 China; 2 Department of Gastrointestinal Surgery, The First Affiliated Hospital, Sun Yat-Sen University, Guangzhou, Guangdong China

**Keywords:** Colon cancer, Anastomotic recurrence, Survival, Resection margin distance

## Abstract

**Purpose:**

Few studies have focused on anastomotic recurrence (AR) in colon cancer. This study aimed to clarify the association of resection margin distance with AR and compare the prognosis with nonanastomotic local recurrence (NAR).

**Methods:**

This retrospective cohort study included the clinical data of patients who underwent radical colon cancer surgery between January 1, 2009, and December 31, 2019.

**Results:**

A total of 1958 colon cancer patients were included in the study. 34 of whom (1.7%) had AR and 105 of whom (5.4%) had NAR. Multivariate analysis revealed that the lower distal resection margin distance, advanced N stage, and number of lymph nodes dissected were risk factors for AR. In the proximal resection margin, the risk of AR was lowest at a distance of 6 cm or greater, with a 3-year rate of 1.3%. In the distal resection margin, the 3-year AR risk increased rapidly if the distance was less than 3 cm. The prognosis of patients in the AR group was similar to that of patients in the NAR group, regardless of synchronous distant metastases. Furthermore, the radical surgery rate for AR was significantly higher than that for NAR, but the prognosis of AR was comparable to that of NAR.

**Conclusions:**

The distal resection margin distance, advanced N stage, and less number of lymph nodes dissected are associated with AR of colon cancer. The prognosis of patients with AR was similar to that of patients with NAR.

**Trial registration:**

Clinical Trial Numbers NCT04074538 (clinicaltrials.gov), August 26, 2019, registered, retrospectively registered.

**Supplementary Information:**

The online version contains supplementary material available at 10.1007/s00384-024-04684-x.

## Introduction

Local recurrence (LR) of colon cancer is often incurable, and the factors associated with it are unclear; only a few studies have attempted to address LR of colon cancer. Anastomotic recurrence (AR) is a particular type of LR of colon cancer, with an incidence of 0.4% to 4.2% [1–3]. Compared with other types of nonanastomotic local recurrence (NAR), AR can be easily diagnosed by colonoscopy biopsy and has a greater opportunity for salvage surgery, but there is no doubt that it is a significant burden for these patients, both physically and economically [4]. The mechanism of AR remains controversial. The dominant theory is that insufficient resection margins, mesangial excision, or implantation of exfoliated tumor cells can cause this complication [5, 6]. This suggests that, unlike other types of nonanastomotic local recurrence, AR may contain residual tumor cells in the resected margin or lymphatic vessels, which requires further research. In addition, colon resection along with en bloc dissection of regional lymph nodes are recommended based on the principle of complete mesocolon excision (CME) according to both the National Comprehensive Cancer Network (NCCN) and the Chinese guidelines [7, 8], but the appropriate distance for surgical margins is not specified. Although CME can reduce the risk of LR [9], quite a few patients with colon cancer still experience AR after CME surgery. One critical influencing factor could be close resection margins, but blindly extending the scope of surgery cannot reduce the risk [10].

The aim of this study was to evaluate the association of resection margin distance with AR and to compare the clinicopathological factors of patients with AR with those with NAR and those who remain local recurrence-free.

## Patients and methods

### Data sources and study design

This was a retrospective population-based study based on a prospectively maintained National Colorectal Cancer Cohort (NCRCC) study database that included patients who were first diagnosed with colorectal cancer between 2009 and 2019. The NCRCC database provides detailed surgery information, demographic data, and pathological characteristics, and the protocol is registered at ClinicalTrials.gov (NCT04074538). The inclusion criteria were as follows: 1) all patients with colon cancer, defined as cancer in the cecum, ascending colon, transverse colon, or sigmoid colon, who underwent radical surgery (negative margin, R0 resection); 2) had pathological stage I to III disease; and 3) had complete follow-up information. The exclusion criteria were as follows: 1) had a tumor proximal edge less than 15 cm to the anal verge (including rectosigmoid junction carcinoma); 2) diagnosed with multiple primary intestinal cancers; 3) underwent urgent or emergency surgery for any reason (obstruction or perforation); 4) received preoperative adjuvant therapy (chemotherapy and/or radiotherapy); 5) diagnosed with hereditary colon cancer or other malignant tumors within 5 years before colon cancer surgery; or 6) died within 90 days following surgery. The study protocol and all amendments were approved by the ethics committee of the Cancer Hospital, Chinese Academy of Medical Sciences and Peking Union Medical College (18–015/1617).

### Outcomes and follow-up

The primary outcomes were LR and survival after recurrence (SAR). LR was defined as recurrence 90 or more days after the first colon curative surgery of a tumor in and around the previous tumor bed, including the pericolic fat, adjoining mesentery, or abdominal lymph nodes, irrespective of location [9]. The curative surgical approach is colectomy plus en bloc dissection of regional lymph nodes according to the CME principle, and only complete resection can be regarded as radical. The root lymph nodes at the origin of the tumor blood vessels and suspected metastatic lymph nodes outside the dissection range should also be removed or biopsied [8]. SAR was defined as the time from the first recurrence to either death or the last follow-up. The surgical margin distance was confirmed according to the pathological specimens. The clinicopathological characteristics (25 variables), including sex, age, history of basic disease, tumor location, preoperative carcinoembryonic antigen (CEA) and carbohydrate antigen 19–9 (CA199) levels, operation duration, bleeding volume, distance to the proximal and distal resection margins, pathological type, morphological type, histological differentiation, lymphatic/vascular invasion, perineural invasion, tumor deposition, number of lymph nodes dissected, T stage and N stage, mismatch repair (MMR) status, and adjuvant therapy, were evaluated. The last follow-up date for the surviving patients was August 2023.

### Identification of AR

**Fig. 1:**
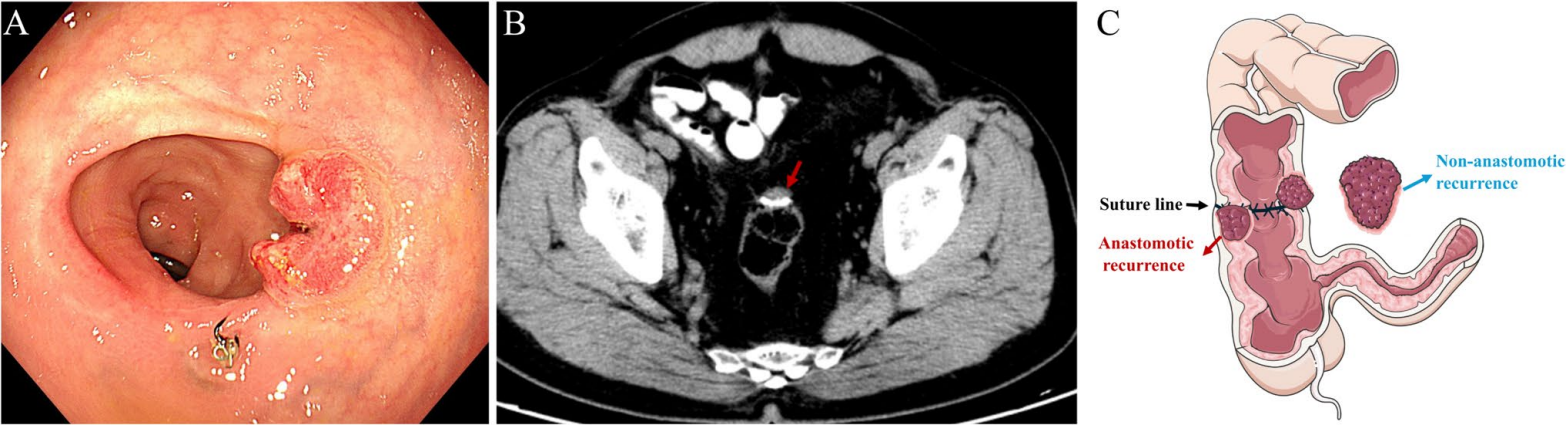
**A** Typical endoscopic images of anastomotic recurrence. **B** Computed tomography images of anastomotic recurrence in the same patient. **C** Schematic diagram of anastomotic recurrence and non-anastomotic recurrence

AR was defined as recurrence in the suture or staple line of the bowel anastomosis, which was pathologically diagnosed by endoscopic biopsy or resected specimen (Fig. 1). Patients with local recurrence other than AR were considered to have NAR. Patients in whom the recurrent tumor was in contact but did not infiltrate the suture line but was located mainly outside the intestinal wall were excluded and considered to have NAR. All patients with LR were stratified into an AR group and an NAR group, and both groups included patients with synchronous distant metastatic recurrence. The remaining patients with no LR were assigned to the control group.

### Statistical analysis

Descriptive variables are presented as medians and interquartile ranges or percentages, as appropriate. The t test for continuous variables and the χ2 test for categorical variables were used to compare factors between groups. For survival analysis, the Kaplan–Meier method was used for comparative analysis with the log-rank test. The time from surgery to recurrence (TTR) was defined as the time between the date of surgery and the date of recurrence. Risk factors for patients with AR and NAR were investigated using univariate and multivariate analyses with Cox proportional hazard models. All *p* values were two-sided, and p values less than 0.05 were considered significant. All the statistical analyses were performed with R software (version 3.6.1). The study adhered to the STROBE reporting recommendation.

## Results

### General characteristics

**Fig. 2:**
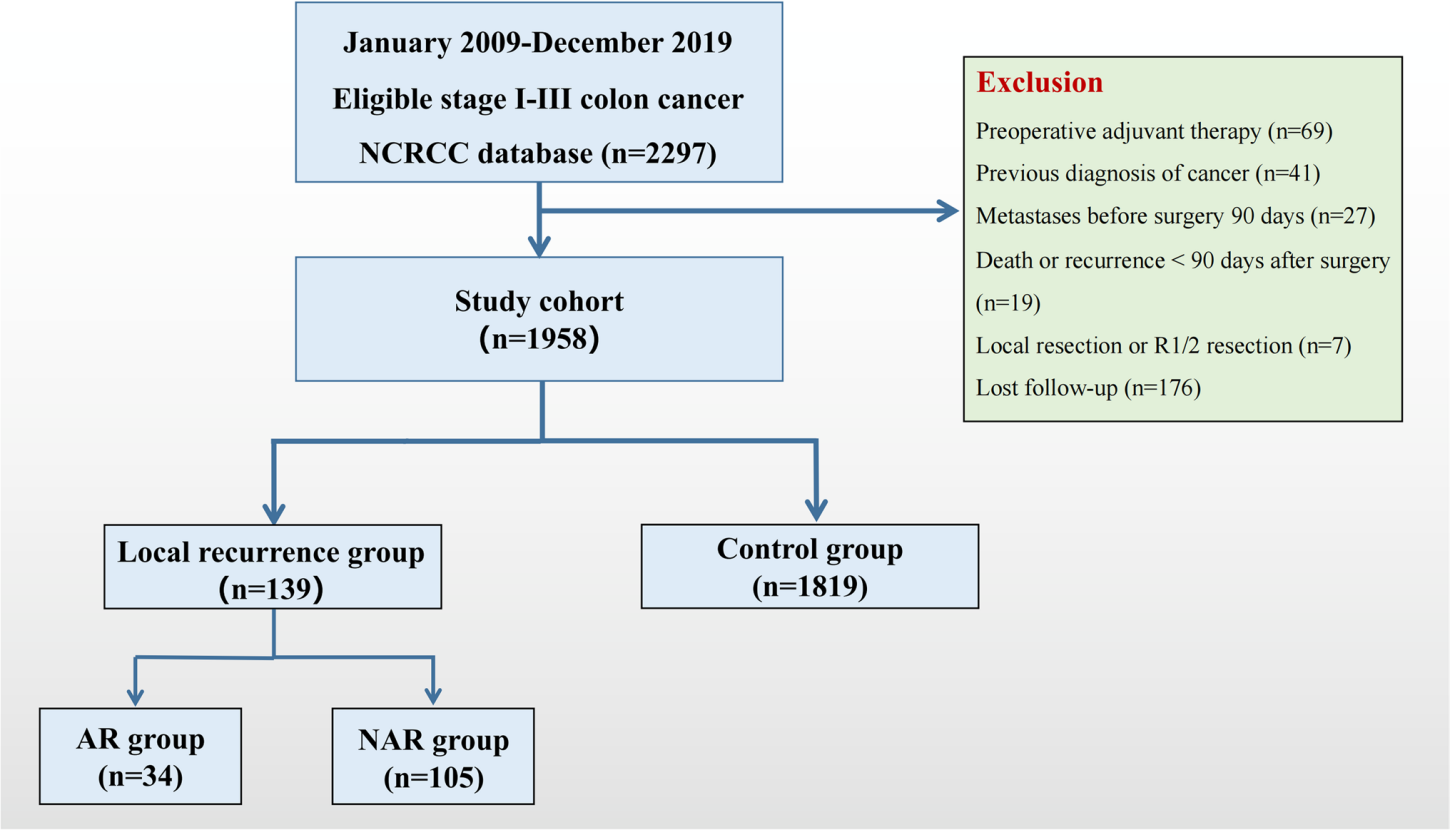
Flow chart showing all enrolled patients. AR = anastomotic recurrence; NAR = non-anastomotic recurrence. NRCRCC = National Colorectal Cancer Cohort

This study included a total of 1958 colon cancer patients who underwent curative surgery (Fig. 2). The median (IQR) follow-up duration was 62 (47–75) months after surgery. Local recurrence was observed in 139 patients (7.1%), 34 of whom (1.7%) had AR and 105 of whom (5.4%) had NAR. Table 1 shows a comparison of the baseline characteristics of the three study groups: the control, AR, and NAR groups. In the comparison between the AR and control groups, the number of lymph nodes dissected was significantly lower in the AR group (*P* < 0.001). The analysis comparing the control group and the NAR group revealed statistically significant differences in bleeding volume, histological differentiation, lymphatic/vascular invasion, perineural invasion, number of lymph nodes dissected, pathological N stage, and postoperative adjuvant therapy (*P* < 0.05). Additionally, the comparison between the AR and NAR groups revealed no significant differences in any of the indicators (*P* > 0.05), indicating that AR has entirely different causes than other types of local recurrence.

**Table 1:** Baseline characteristics of each group

Characteristics	Control (*N* = 1819), n%	AR (*N* = 34), n%	NAR (*N* = 105), n%	*P* value^a^	*P* value^b^	*P* value^c^
Age, (years, mean ± SD)	60.04 ± 11.60	60.05 ± 11.23	61.76 ± 11.21	0.788	0.140	0.598
Sex				0.254	0.561	0.470
Male	1109 (61.0)	24 (70.6)	67 (63.8)			
Female	710 (39.0)	10 (29.4)	38 (36.2)			
BMI, (kg/m^2^,mean ± SD)	23.18 ± 4.79	23.24 ± 5.07	23.73 ± 4.41	0.946	0.263	0.609
History of diabetes				0.158	0.061	0.579
Missing	509	15	32			
No	1109 (84.7)	18 (94.7)	68 (93.2)			
Yes	201 (15.3)	1 (5.3)	5 (6.8)			
History of cardiovascular diseases				0.389	0.722	0.339
Missing	509	15	32			
No	1200 (91.6)	18 (94.7)	66 (90.4)			
Yes	110 (8.4)	1 (5.3)	7 (9.6)			
History of gastroenteritis				0.726	0.633	0.904
Missing	506	17	32			
No	1122 (85.6)	14 (82.4)	61 (83.6)			
Yes	189 (14.4)	3 (17.6)	12 (16.4)			
Tumor sidedness				0.172	0.427	0.578
Right colon	1046 (57.5)	25 (73.5)	67 (63.7)			
Transverse	89 (4.9)	1 (2.9)	5 (4.9)			
Left colon	684 (37.6)	8 (23.5)	33 (31.4)			
Preoperative CEA, (ng/ml, mean ± SD)	8.81 ± 22.29	10.31 ± 20.22	17.11 ± 43.61	0.719	0.070	0.419
Preoperative CA199, (IU/mL, mean ± SD)	25.44 ± 62.15	25.60 ± 57.03	42.92 ± 104.58	0.985	0.120	0.422
Operation duration, (mins, mean ± SD)	149.3 ± 50.98	168.72 ± 54.32	160.47 ± 56.54	0.110	0.055	0.585
Bleeding volume, (ml, mean ± SD)	55.76 ± 86.71	83.33 ± 134.76	92.95 ± 155.38	0.186	0.040	0.809
Hospital day, (days, mean ± SD)	8.55 ± 2.80	9.94 ± 5.54	8.83 ± 2.77	0.159	0.316	0.126
Proximal resection margin distance, (cm, mean ± SD)	8.56 ± 5.29	8.59 ± 6.31	8.94 ± 5.14	0.965	0.468	0.749
Distal resection margin distance, (cm, mean ± SD)	4.35 ± 2.91	3.85 ± 3.59	4.81 ± 3.40	0.323	0.118	0.161
Pathological type				0.380	0.421	0.771
Adenocarcinoma	1638 (90.0)	29 (85.3)	92 (87.6)			
Mucinous/signet	181 (10.0)	5 (14.7)	13 (12.4)			
Tumor morphological type				0.620	0.308	0.935
Missing	192	3	8			
Protruded	808 (49.7)	14 (45.2)	43 (44.3)			
Ulcerative/infiltrating	819 (50.3)	17 (54.8)	54 (55.7)			
Histological differentiated				0.632	0.005	0.339
Missing	56	1	2			
Moderate/Highly	1243(70.5)	22 (66.7)	59 (57.0)			
Poor	520 (29.5)	11 (33.3)	44 (43.0)			
Lymphatic/vascular invasion				0.321	< 0.001	0.386
Missing	100	6	7			
No	1250 (72.7)	18 (64.3)	54 (55.1)			
Yes	469 (27.3)	10 (35.7)	44 (44.9)			
Perineural invasion				0.399	0.004	0.534
Missing	111	6	8			
No	1222 (71.5)	18 (64.3)	56 (57.7)			
Yes	486 (28.5)	10 (35.7)	41 (42.3)			
Tumor deposit				0.742	0.241	0.735
No	1685 (92.6)	32 (94.1)	94 (89.5)			
Yes	134 (7.4)	2 (5.9)	11 (10.5)			
Number of lymph nodes dissection, (mean ± SD)	27.39 ± 13.60	21.18 ± 8.89	24.34 ± 12.54	< 0.001	0.026	0.171
Pathological T stage				0.422	0.598	0.735
T1/2	222 (12.2)	2 (5.9)	11 (10.5)			
T3/4	1598 (87.8)	32 (94.1)	94 (89.5)			
Pathological N stage				0.235	< 0.001	0.346
N0	1244 (68.4)	20 (58.8)	52 (49.5)			
N1/2	575 (31.6)	14 (41.2)	53 (51.5)			
MMR status				0.790	0.437	0.848
Missing	55	3	4			
pMMR	1524 (86.4)	28 (90.3)	90 (89.1)			
dMMR	240 (13.6)	3 (9.7)	11 (10.9)			
Postoperative adjuvant therapy				0.365	0.016	0.668
No	998 (54.8)	16 (47.1)	45 (42.9)			
Yes	821 (45.2)	18 (52.9)	60 (57.1)			

*Abbreviation*: *BMI* body mass index, *CEA* carcinoembryonic antigen, *CA199* Carbohydrate antigen 199, *dMMR* deficient mismatch repair, *MMR* mismatch repair

^a^*P* value Control vs. AR

^b^*P* value Control vs. NAR

^c^*P* value AR vs. NAR

### Univariate and multivariate analyses of AR and NAR

Cox proportional hazard models were used to analyze the risk factors for AR in all cohorts (Table 2). Multivariate analysis revealed significant differences in the number of distal resection margin distance (DRMD) (HR 2.40, 95% CI: 1.06–5.42, *P* = 0.035), pathological N stage (HR 2.49, 95% CI: 1.15–5.39, *P* = 0.021) and the number of lymph nodes dissected (HR 0.95, 95% CI: 0.91–0.99, *P* = 0.025). However, the risk of AR was not significantly different between patients with a lower proximal resection margin distance (PRMD) (*P* = 0.426). We also analyzed the risk factors for NAR in all cohorts (Table 3). Multivariate analysis revealed significant differences in CA199 (HR 1.81, 95% CI: 1.06–3.09, *P* = 0.029), the number of lymph nodes dissected (HR 0.97, 95% CI: 0.95–0.99, *P* = 0.008), and pathological N1/2 stage (HR 1.76, 95% CI: 1.04–2.97, *P* = 0.034).

**Table 2:** Univariate and multivariable analysis for anastomotic recurrence (AR) in all cohorts

Characteristics	Univariate		Multivariable	
	HR (95%CI)	*P*	HR (95%CI)	*P value*
Sex (male)	0.58 (0.25–1.39)	0.224		
Age (≥ 70 y)	0.96 (0.36–2.56)	0.942		
BMI (≥ 25 kg/m^2^)	0.93 (0.38–2.28)	0.870		
History of diabetes (yes)	0.04 (0.01–117.74)	0.429		
History of cardiovascular diseases (yes)	0.04 (0.01–140.40)	0.554		
History of gastroenteritis (yes)	1.67 (0.35–8.03)	0.523		
Preoperative CEA (≥ 5 ng/mL)	1.20 (0.50–2.89)	0.689		
Preoperative CA199 (≥ 37 IU/mL)	1.30 (0.38–4.47)	0.674		
Proximal resection margin distance (cm)	1.37 (0.63–2.95)	0.426		
Distal resection margin distance (cm)	2.80 (1.25–6.29)	0.012	2.40 (1.06–5.42)	0.035
Operation duration (≥ 180 min)	1.35 (0.35–5.20)	0.667		
Bleeding volume (ml)	1.00 (0.99–1.01)	0.121		
Pathological type (mucinous/signet)	2.07 (0.78–5.50)	0.143		
Tumor morphological type (ulcer/infiltrate)	1.16 (0.52–2.59)	0.720		
Histological differentiated (poor)	1.39 (0.60–3.05)	0.475		
Lymphatic/vascular invasion (positive)	1.73 (0.71–4.24)	0.229		
Perineural invasion (positive)	1.64 (0.67–4.02)	0.277		
Tumor deposit (positive)	1.06 (0.26–4.61)	0.933		
Number of lymph nodes dissected	0.94 (0.90–0.99)	0.009	0.95 (0.91–0.99)	0.025
Pathological T stage (T4)	1.20 (0.55–2.62)	0.643		
Pathological N stage (N1/2)	2.57 (1.19–5.55)	0.017	2.49 (1.15–5.39)	0.021
Mismatch repair status (dMMR)	0.60 (0.14–2.56)	0.490		
Postoperative adjuvant therapy	1.34 (0.62–2.89)	0.460		

*Abbreviation*: *BMI* body mass index, *CEA* carcinoembryonic antigen, *CA199* Carbohydrate antigen 199, *ulcer* ulcerative, *dMMR* deficient mismatch repair

**Table 3:** Univariate and multivariable analysis for non-anastomotic recurrence (NAR) in all cohorts

Characteristics	Univariate		Multivariable	
	HR (95%CI)	*P*	HR (95%CI)	*P value*
Sex (male)	0.89 (0.60–1.33)	0.580		
Age (≥ 70 y)	1.39 (0.90–2.16)	0.139		
BMI (≥ 25 kg/m^2^)	1.23 (0.81–1.85)	0.336		
History of diabetes (yes)	0.42 (0.17–1.04)	0.061		
History of cardiovascular diseases (yes)	1.16 (0.53–2.53)	0.706		
History of gastroenteritis (yes)	1.15 (0.62–2.13)	0.664		
Preoperative CEA (≥ 5 ng/mL)	1.15 (0.76–1.75)	0.511		
Preoperative CA199 (≥ 37 IU/mL)	2.14 (1.31–3.48)	0.002	1.81 (1.06–3.09)	0.029
Proximal resection margin distance (cm)	0.86 (0.58–1.28)	0.454		
Distal resection margin distance (cm)	0.95 (0.64–1.41)	0.806		
Operation duration (≥ 180 min)	1.65 (1.06–2.56)	0.079		
Bleeding volume (ml)	0.47 (1.12–1.90)	0.289		
Pathological type (mucinous/signet)	1.08 (0.60–1.94)	0.794		
Tumor morphological type (ulcer/infiltrate)	1.23 (0.82–1.84)	0.316		
Histological differentiated (poor)	1.91 (1.29–2.83)	0.001	1.53 (0.96–2.45)	0.077
Lymphatic/vascular invasion (positive)	2.33 (1.56–3.48)	< 0.001	1.50 (0.89–2.54)	0.128
Perineural invasion (positive)	1.87 (1.25–2.80)	0.002	1.09 (0.67–1.80)	0.722
Tumor deposit (positive)	1.67 (0.89–3.13)	0.109		
Number of lymph nodes dissected	0.98 (0.97–1.00)	0.027	0.97 (0.95–0.99)	0.008
Pathological T stage (T4)	1.76 (1.20–2.59)	0.004	1.23 (0.77–1.96)	0.399
Pathological N stage (N1/2)	2.68 (1.80–3.98)	< 0.001	1.76 (1.04–2.97)	0.034
Mismatch repair status (dMMR)	0.75 (0.400–1.40)	0.359		
Postoperative adjuvant therapy	1.52 (1.03–2.24)	0.033	1.10 (0.69–1.76)	0.692

*Abbreviation BMI* body mass index, *CEA* carcinoembryonic antigen, *CA199* Carbohydrate antigen 199, *ulcer* ulcerative, *dMMR* deficient mismatch repair

### Association of surgical margin distance with AR

Although the distance from the tumor to the proximal margin in patients was not significantly different between the AR groups, the average proximal resection margin distances were lower in the AR group, as shown in Table 1 (proximal: 8.59 cm vs. 8.94 cm, *P* = 0.749). Therefore, we analyzed the association between surgical margin distance (PRMD and DRMD) and AR in all cohorts. Table 4 shows the AR and NAR rates for different surgical margin distance cutoff values in pathological specimens, separated by proximal vs. distal. In the proximal resection margin, the risk of AR was lowest at a distance of 6 cm or greater, with 3-year and 5-year rates of 1.3% and 1.4%, respectively. However, patients with a PRMD greater than 6 cm did not have a lower AR rate (Supplementary Figure 1A). In the distal resection margin, the 3-year AR risk increased rapidly if the distance was less than 3 cm, and the AR risk was significantly greater in patients with a DRMD less than 3 cm (Supplementary Figure 1B). In addition, the 3-year and 5-year rates of NAR did not vary with resection margin distance.

**Table 4:** Anastomotic recurrence (AR) rates and non-anastomotic recurrence (NAR) rates for different cutoff values for surgical margin distance

	Proximal resection margin distance (PRMD)					Distal resection margin distance (DRMD)				
		AR, %		NAR, %			AR, %		NAR, %	
Characteristics	No. (n%)	3-y rate	5-y rate	3-y rate	5-y rate	No. (n%)	3-y rate	5-y rate	3-y rate	5-y rate
1 cm										
≤ 1 cm	1 (0.0)	0	0	0	0	96 (4.9)	3.1	4.2	2.1	5.2
1 cm	1957 (100.0)	1.5	1.7	4.2	4.8	1862 (95.1)	1.5	1.6	4.3	4.9
2 cm										
≤ 2 cm	21 (1.1)	9.5	9.5	0	4.8	381 (19.5)	2.4	2.9	2.4	2.9
2 cm	1937 (98.9)	1.4	1.6	4.2	4.8	1577 (80.5)	1.3	1.4	4.6	5.3
3 cm										
≤ 3 cm	87 (4.4)	4.6	4.6	3.4	3.4	792 (40.4)	2.1	2.5	3.9	4.5
3 cm	1871 (95.6)	1.4	1.5	4.2	4.9	1166 (59.6)	1.1	1.1	4.4	5.0
4 cm										
≤ 4 cm	257 (13.1)	2.3	3.1	3.1	3.5	1215 (62.1)	1.8	2.1	4.0	4.6
4 cm	1701 (86.9)	1.4	1.5	4.4	5.0	743 (37.9)	1.1	1.1	4.6	5.1
5 cm										
≤ 5 cm	559 (28.5)	1.8	2.1	3.8	4.7	1472 (75.2)	1.6	1.8	3.9	4.6
5 cm	1399 (71.5)	1.4	1.5	4.4	4.9	486 (24.8)	1.2	1.2	5.1	5.6
6 cm										
≤ 6 cm	823 (42.0)	1.8	2.1	3.9	4.5	1627 (83.1)	1.6	1.8	3.7	4.4
6 cm	1135 (58.0)	1.3	1.4	4.4	4.9	331 (16.9)	1.2	1.2	6.6	6.9
7 cm										
≤ 7 cm	1042 (53.2)	1.5	1.7	3.9	4.7	1728 (88.3)	1.6	1.7	3.6	4.3
7 cm	916 (46.8)	1.5	1.6	4.5	4.9	230 (11.7)	1.3	1.3	8.2	8.2
8 cm										
≤ 8 cm	1181 (60.3)	1.5	1.7	3.6	4.4	1807 (92.3)	1.5	1.7	3.9	4.6
8 cm	777 (39.7)	1.5	1.7	5.0	5.3	151 (7.7)	1.9	1.9	7.3	7.3
9 cm										
≤ 9 cm	1316 (67.2)	1.5	1.7	3.7	4.5	1850 (94.5)	1.5	1.6	3.9	4.6
9 cm	642 (32.8)	1.6	1.7	5.1	5.5	108 (5.5)	2.8	2.8	8.3	8.3
10 cm										
≤ 10 cm	1446 (73.9)	1.5	1.7	3.9	4.6	1881 (96.1)	1.4	1.6	4.0	4.7
10 cm	512 (26.1)	1.6	1.8	5.1	5.3	77 (3.9)	3.9	3.9	7.8	7.8

*Abbreviation*: *AR* anastomotic recurrence, *NAR* non-anastomotic recurrence

### Treatment of AR and NAR

Radical surgery was performed on 76.5% (26/34) of the AR patients and 28.6% (30/105) of the NAR patients (Table 5). Adjuvant therapy was administered to 14.7% and best supportive care to 5.9% of the AR patients. However, approximately half of the patients with NAR (49.6%) received adjuvant therapy, and 20.0% received only best supportive care. Among all patients with LR (AR patients and NAR patients), 10 (18.1%), 62 (44.6%), and 67 (48.2%) had primary stage I, stage II, and stage III disease, respectively. Interestingly, a higher proportion of LR patients with primary tumor stage I and II recurrence underwent radical surgery than patients with LR with primary tumor stage III (54.2% vs. 25.4%, *P* < 0.001), which suggests that stage III tumors are more aggressive than stage I and II tumors.

**Table 5:** Treatment of patients with anastomotic recurrence (AR) and non-anastomotic recurrence (NAR)

	AR, *N* = 34, (%)				NAR, *N* = 105, (%)			
Characteristics	No	Stage I^*^	Stage II^*^	Stage III^*^	No	Stage I^*^	Stage II^*^	Stage III^*^
Surgical treatment								
Radical surgery	26 (76.5)	5.9	44.1	26.5	30 (28.6)	5.7	15.2	7.6
Palliative surgery	1 (2.9)	0	0	2.9	2 (1.9)		1.0	1.0
Adjuvant therapy								
Chemotherapy	4 (11.8)	0	5.9	5.9	30 (28.6)	1.9	11.4	15.2
Chemotherapy plus targeted therapy	1 (2.9)	0	0	2.9	21 (20.0)	0	5.7	14.3
Immunotherapy	0	0	0	0	1 (1.0)			
Best supportive care	2 (5.9)	0	2.9	2.9	21 (20.0)	0	7.6	12.4

*Abbreviation AR* anastomotic recurrence, *NAR* non-anastomotic recurrence

^*^Pathological stage of primary surgery

### Survival with AR and NAR

**Fig. 3:**
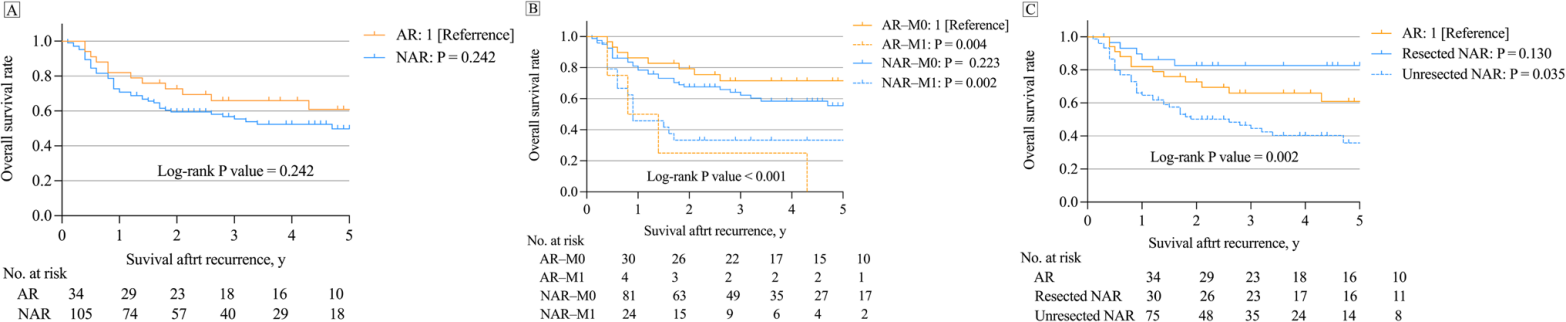
Survival after recurrence curves for all local recurrence patients. **A** AR and NAR group. **B** The AR and NAR groups were stratified into 2 groups with or without synchronous distant metastases. **C** The NAR groups were stratified into 2 groups with or without resected. AR = anastomotic recurrence; NAR = non-anastomotic recurrence. AR-M0 = AR without synchronous distant metastases; AR-M1 = AR with synchronous distant metastases; NAR-M0 = NAR without synchronous distant metastases; NAR-M1 = NAR with synchronous distant metastases

The median SAR times in the AR and NAR groups were 3.2 years and 2.2 years, respectively, irrespective of synchronous distant metastases (within 6 months before or after the diagnosis of LR) and treatment. The AR group tended to have a better prognosis than the NAR group had (3-y SAR: AR 66.0% vs. NAR 55.4%; *P* = 0.242) (Fig. 3A). Further subgroup analysis of patients with or without synchronous distant metastases revealed that patients in the AR-M1 and NAR-M1 groups had a significantly poorer prognosis, whereas those in the AR-M0 group had a prognosis comparable to that of patients in the NAR-M0 group (3-y SAR: AR-M0 71.5% vs. NAR-M0 64.1%, *P* = 0.223) (Fig. 3B), the number and the details of treatments of patients who developed synchronous distant metastases was shown in Supplementary Table 1. When the NAR patients were classified into resected (radical surgery) and unresected groups, the unresected NAR group had a significantly poorer prognosis, whereas the resected NAR group had a prognosis comparable to that of the AR group (3-year SAR: AR 66.0% vs. resected NAR 82.6%, *P* = 0.130; AR 66.0% vs. unresected NLR 44.6%, *P* = 0.035) (Fig. 3C).

## Discussion

Few studies have investigated the association between resection margin distance and AR of colon cancer, especially when distinguishing between AR and other LR types and comparing them within the same cohort. These findings suggest that PRMD is not an independent risk factor for AR, whereas lower DRMD, fewer lymph nodes dissected, and advanced N stage are significantly correlated with AR. Compared to AR, NAR has more complex causes and more risk factors. A DRMD of 3 cm or greater on pathological specimens to 6 cm or greater on PRMD significantly had the lowest risk of AR at 3 years. In addition, the radical surgery rate in the AR group was significantly higher than that in the NAR group, and those patients had a better SAR.

The mechanisms of anastomotic recurrence are complex, and most clinicians would attribute its occurrence to unradical resection margins or insufficient resection margin distance. Our findings showed a significant difference between DRMD and AR instead of PRMD, which is consistent with the consensus in clinical practice. In theory, AR can be caused by the implantation of intraluminal exfoliated tumor cells into the suture line or metastasis of tumor cells before resection without detection of distant micrometastases on the preoperative imaging scan [11, 12]. The distance from the tumor is inversely correlated with the number of exfoliated tumor cells in the intestine [13]. Bai F et al. reported a large amount of mutation accumulation and clonal expansion in the normal-morphologically normal epithelial tissue adjacent to the tumor [14], which may lead to anastomotic recurrence. However, we found that the average proximal and distal resection margin distances were lower in the AR group, and a DRMD of 3 cm or greater was considered a safe resection margin, maintaining the risk of 3-year AR at 1.1%. Many studies have shown that extensive resection results in a higher number of harvested lymph nodes but does not prevent the development of local recurrence, indicating the importance of tumor biology [9, 15]. This finding is in concordance with our findings that even though the 3-year AR rate was lowest when the PRMD was greater than 6 cm, there was still no significant difference in the AR rate. Moreover, previous studies have shown that advanced N stage and large tumor size may determine rectal AR [3, 16]. In colon cancer, little is known about the exact reason for AR after radical surgery with curative intent. Few studies have shown that CEA, mucinous differentiation, and lymphovascular invasion are independent risk factors for AR [2, 17]. Our results showed that the advanced N stage and the number of lymph nodes dissected were risk factors for AR, but the advanced T stage was not associated with AR. Compared with patients with colon cancer, rectal cancer patients are more likely to undergo tumor manipulation due to anatomical confinement of the narrow pelvic cavity and considerably shorter DRMD [18]. Therefore, the more advanced the N and T stages are, the greater the chances of intraluminal seeding of exfoliated tumor cells. A greater number of lymph nodes dissected means more extensive removal of lymph nodes harboring isolated tumor cells or micrometastases, which could impact survival by causing LR or even distant metastases [19, 20]. The fundamental reason for fewer lymph nodes retrieved is insufficient mesenteric resection, which may result in failure to resect all positive lymph nodes, and thus results in AR. However, this still requires further verification in large prospective cohort studies.

The second main finding from this study was that the SAR times were similar in both the AR and NAR groups, regardless of synchronous distant metastases. Furthermore, the prognosis of patients in the AR group was comparable to that of patients in the NAR group. This is another difference from rectal cancer. The OS time of patients with AR of rectal cancer was longer than that of patients with pelvic cancer [21]. We propose that the main reason for this difference is that LR in rectal cancer patients can easily invade the pelvic cavity and cause serious complications such as obstruction, whereas NAR of colon cancer is mainly associated with lymph node recurrence (mesenteric/nodal, retroperitoneal), which is less likely to cause symptoms of obstruction and perforation. R0 resection is the decisive factor in the long-term survival of patient with LR of both colon cancer and rectal cancer [22, 23], which is in concordance with our findings that compared to the unresected NAR group, the AR group had a significantly better prognosis. Furthermore, surveillance colonoscopy is very important, as we detected some early recurrences that were not obvious on imaging but could be easily diagnosed and even cured under colonoscopy, and there were also patients who experienced 2 recurrences up to 5 years after surgery. Previous reports revealed that neoadjuvant chemotherapy and multivisceral resection are feasible treatment options for AR [24]. In our study, there was no evidence for the usefulness of adjuvant chemotherapy after LR, including AR. Therefore, early detection by strict surveillance and curative resection is recommended to optimize the prognosis of AR patients.

The strengths of the current study are the large cohort and detailed clinicopathological characteristics, which increase the statistical power and confidence in the reported recurrence rates; moreover, considerable time and resources are required to obtain follow-up data from medical records. One of the limitations of this study is that it was a single-center study. Second, as we focused on accurately assessing the incidence of AR, patients who underwent proctosigmoidectomy and patients who received preoperative radiation therapy or chemoradiation therapy were excluded. Third, the assessment may miss or misdiagnose a recurrence. In some patients, recurrence can be identified only by reviewing outpatient medical records, which usually lack imaging and pathology results.

Despite the abovementioned unavoidable limitations, it is believed that this study will lead to further research into the individualized management of AR of colon cancer. This population-based registry study revealed that the number of lymph nodes dissected and length of hospital stay were associated with AR of colon cancer. The resection margin distance from the tumor is important and may be the key to making clinical decisions. The radical surgery rate for AR was significantly higher than that for NAR, but the survival outcomes after recurrence were comparable. These data may provide insight into the optimal resection margin for preventing AR after colon cancer surgery.

## Supplementary Information

Below is the link to the electronic supplementary material.

Supplementary Material 1: Supplement Figure 1 Association of resection margin distance with anastomotic recurrence. A, PRMD cut-off value is 6cm. B, DRMD cut-off value is 3cm. PRMD = proximal resection margin distance; DRMD = distal resection margin distance.

Supplementary Material 2: Supplementary Table 1. Statistics of patients with synchronous distant metastasis of anastomotic recurrence (AR) and non-anastomotic recurrence (NAR)

## Data Availability

No datasets were generated or analysed during the current study.
