# Supplementary figures and images for: Association of resection margin distance with anastomotic recurrence in stage I-III colon cancer: data from the National Colorectal Cancer Cohort (NCRCC) study in China
Source: Int J Colorectal Dis. 2024 Jul 12;39(1):105. doi: 10.1007/s00384-024-04684-x (PMC11245431; doi:10.1007/s00384-024-04684-x)

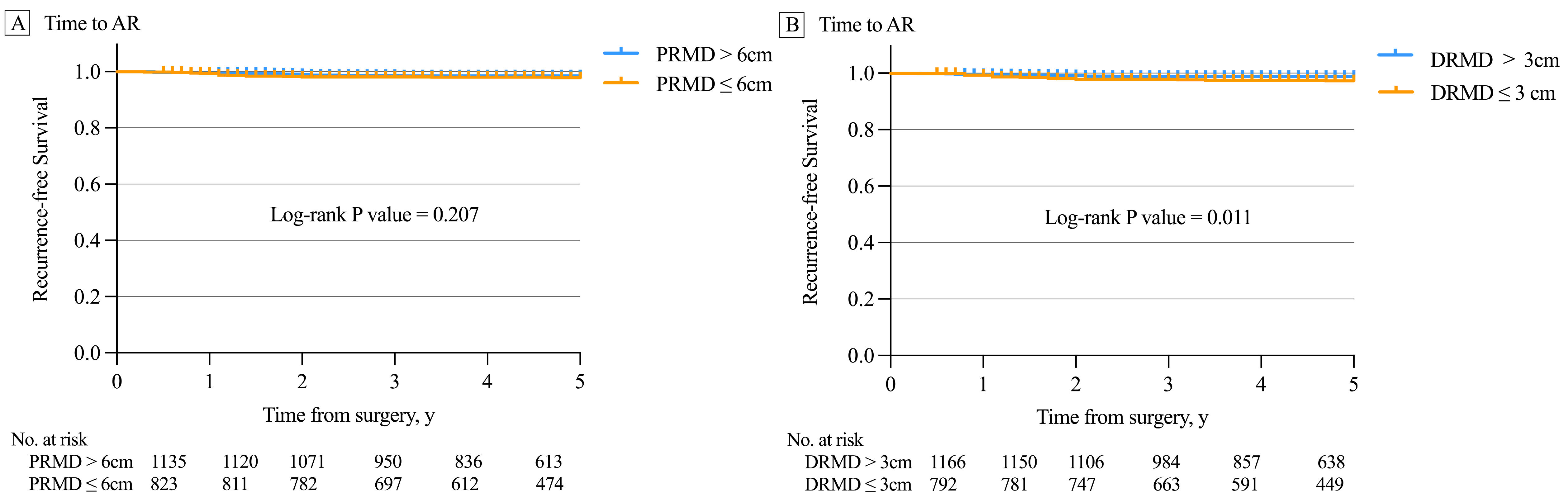

Supplement: Supplementary file 1 — Supplementary Material 1: Supplement Figure 1 Association of resection margin distance with anastomotic recurrence. A, PRMD cut-off value is 6cm. B, DRMD cut-off value is 3cm. PRMD = proximal resection margin distance; DRMD = distal resection margin distance. [file 384_2024_4684_MOESM1_ESM.tif]
