# Supplementary material for: Association of resection margin distance with anastomotic recurrence in stage I-III colon cancer: data from the National Colorectal Cancer Cohort (NCRCC) study in China
Source: Int J Colorectal Dis. 2024 Jul 12;39(1):105. doi: 10.1007/s00384-024-04684-x (PMC11245431; doi:10.1007/s00384-024-04684-x)
Supplement: Supplementary file 2 — Supplementary Material 2: Supplementary Table 1. Statistics of patients with synchronous distant metastasis of anastomotic recurrence (AR) and non-anastomotic recurrence (NAR) [file 384_2024_4684_MOESM2_ESM.docx]

**Supplementary Table 1. Statistics of patients with synchronous distant metastasis of anastomotic recurrence (AR) and non-anastomotic recurrence (NAR)**

| Characteristics | AR  (N=34), n% | NAR  (N=105), n% | *P* value |
| --- | --- | --- | --- |
| Synchronous distant metastases |  |  |  |
| Total | 4 (11.8) | 24 (22.9) | 0.220 |
| Liver only | 1 (2.9) | 5 (4.8) |  |
| Lung only | 0 | 2 (1.9) |  |
| Brain only | 1 (2.9) | 0 |  |
| Multiple metastases (at least 2 organs) | 2 (5.9) | 10 (9.5) |  |
| Other locations | 0 | 7 (6.7) |  |
| Treatment of synchronous distant metastases |  |  |  |
| Radical surgery | 0 | 1 (1.0) |  |
| Chemotherapy | 1 (2.9) | 10 (9.5) |  |
| Chemotherapy plus targeted therapy | 1 (2.9) | 8 (7.6) |  |
| Immunotherapy | 0 | 0 |  |
| Best supportive care | 2 (5.9) | 5 (4.8) |  |

Abbreviation: AR, anastomotic recurrence; NAR, non-anastomotic recurrence.
